# Establishment of Highly Tumorigenic Human Colorectal Cancer Cell Line (CR4) with Properties of Putative Cancer Stem Cells

**DOI:** 10.1371/journal.pone.0099091

**Published:** 2014-06-12

**Authors:** Rebecca A. Rowehl, Stephanie Burke, Agnieszka B. Bialkowska, Donald W. Pettet, Leahana Rowehl, Ellen Li, Eric Antoniou, Yuanhao Zhang, Roberto Bergamaschi, Kenneth R. Shroyer, Iwao Ojima, Galina I. Botchkina

**Affiliations:** 1 Department of Pathology, Stony Brook University Medical Center, Stony Brook, New York, United States of America; 2 Department of Medicine, Stony Brook University Medical Center, Stony Brook, New York, United States of America; 3 Genome Research Center, Cold Spring Harbor Laboratory, Cold Spring Harbor, New York, United States of America; 4 Center of Excellence in Wireless and Information Technology (CEWIT), Stony Brook University, Stony Brook, New York, United States of America; 5 Department of Surgery/Surgical Oncology, Stony Brook University Medical Center, Stony Brook, New York, United States of America; 6 Institute of Chemical Biology and Drug Discovery, Stony Brook University, Stony Brook, New York, United States of America; 7 Department of Chemistry, Stony Brook University, Stony Brook, New York, United States of America; Southern Illinois University School of Medicine, United States of America

## Abstract

**Background:**

Colorectal cancer (CRC) has the third highest mortality rates among the US population. According to the most recent concept of carcinogenesis, human tumors are organized hierarchically, and the top of it is occupied by malignant stem cells (cancer stem cells, CSCs, or cancer-initiating cells, CICs), which possess unlimited self-renewal and tumor-initiating capacities and high resistance to conventional therapies. To reflect the complexity and diversity of human tumors and to provide clinically and physiologically relevant cancer models, large banks of characterized patient-derived low-passage cell lines, and especially CIC-enriched cell lines, are urgently needed.

**Principal Findings:**

Here we report the establishment of a novel CIC-enriched, highly tumorigenic and clonogenic colon cancer cell line, CR4, derived from liver metastasis. This stable cell line was established by combining 3D culturing and 2D culturing in stem cell media, subcloning of cells with particular morphology, co-culture with carcinoma associated fibroblasts (CAFs) and serial transplantation to NOD/SCID mice. Using RNA-Seq complete transcriptome profiling of the tumorigenic fraction of the CR4 cells in comparison to the bulk tumor cells, we have identified about 360 differentially expressed transcripts, many of which represent stemness, pluripotency and resistance to treatment. Majority of the established CR4 cells express common markers of stemness, including CD133, CD44, CD166, EpCAM, CD24 and Lgr5. Using immunocytochemical, FACS and western blot analyses, we have shown that a significant ratio of the CR4 cells express key markers of pluripotency markers, including Sox-2, Oct3/4 and c-Myc. Constitutive overactivation of ABC transporters and NF-kB and absence of tumor suppressors p53 and p21 may partially explain exceptional drug resistance of the CR4 cells.

**Conclusions:**

The highly tumorigenic and clonogenic CIC-enriched CR4 cell line may provide an important new tool to support the discovery of novel diagnostic and/or prognostic biomarkers as well as the development of more effective therapeutic strategies.

## Introduction

Colorectal cancer has the third highest incidence and mortality rate among the US population [1]. The current lack of curative chemotherapies and the highest attrition rate of anticancer drugs compared to other diseases (only 5% of agents that have anticancer activity in preclinical development are licensed; [2]) create an urgent need for more physiologically and clinically relevant sources of cancer cells, as well as for more relevant *in vitro* and *in vivo* models. Traditional cancer research and preclinical evaluation of candidate anticancer agents are based on the use of unselected long-term, high-passage established cancer cell lines grown as a monolayer cultures. However, long-term *in vitro* maintenance inevitably leads to the accumulation of additional genomic and epigenomic changes, as well as the selection of dominant cell subpopulations. Indeed, it was recently demonstrated that the most commonly used established cancer cell lines have no correlation with original clinical samples [3]. This suggests that the use of established cell lines for the study of genomic alterations, discovery of clinically relevant molecular targets, and anticancer drug development is questionable, since the use of these cell lines does not account for the complexity and pathophysiology of *in vivo* tumors.

It is largely accepted now that human tumors are organized hierarchically, and the top of this hierarchy is occupied by malignant stem cells, which possess unlimited self-renewal and tumor­-initiating capacities. According to the most recent concept of carcinogenesis, which has revolutionized the understanding of tumorigenesis and cancer treatment, only specific phenotypic subpopulation(s) of cancer stem cells (CICs) or cancer-initiating cells (CICs) are responsible for tumor development, production of the entire spectrum of the differentiated progeny that compose a tumor mass, metastasis, and resistance to anti-cancer therapies [4]–[6]. Such cells were recently isolated from all major human cancer types, including colorectal cancers [7]–[9]. Numerous studies have demonstrated that specific phenotypes of stem-like tumor-initiating cancer cells are highly drug resistant and are capable of self-renewal after standard therapeutic interventions [10]–[17]. All of the above considerations highlight the crucial role of CICs in the discovery of clinically relevant molecular targets and anticancer drug development.

The identification and characterization of patient-derived CICs, the development of optimal *in vivo* and *in vitro* preclinical models, and CIC-targeted analyses of drug-induced alterations represent critical steps in the assessment of novel anti-cancer therapies. It is evident also that in order to maintain suitable fidelity to the original tumors, the cancer-initiating cells (CICs), as well as other cell types used for genomic and proteomic profiling, should be isolated from a large spectrum of primary and metastatic tumors, not from the established cancer cell lines. However, it is notoriously difficult to establish primary cell lines and particularly CIC lines from fresh tumor specimens [18]. First, there are objective difficulties in the isolation of pure cell populations from heterogeneous tumor tissues. Tumor impurity (different levels of non-tumor cell contamination) and multiclonality are well-documented problems [19], [20]. At the molecular level, there are currently no definitive markers to prove the malignant or nonmalignant nature of cells, as well as to accurately distinguish between normal and cancer stem cells. Growing evidence also suggests that CICs might represent a heterogeneous subpopulation of the tumor-initiating cells [21]–[25]. Nevertheless, a combination of multiple approaches and multiple cell surface markers followed by a thorough functional characterization of the isolated cell phenotypes may enable the purification of the most functionally significant, i.e. tumor-and metastasis-initiating and the most drug-resistant cells. Several colorectal cell lines of different cellular, biochemical and molecular characteristics have been established during the last two decades [26]–[30]. Here we report the establishment of a novel CIC-enriched, highly tumorigenic colorectal cancer cell line isolated from liver metastasis of a CRC patient.

## Results

### Patient specimens and primary cultures

Dissociated cell suspensions from 13 freshly resected colorectal carcinomas of various histological grades and 3 liver metastases were tested for clonogenic and tumorigenic potential *in vivo* and *in vitro* as described in the Methods section. Two specimens were severely contaminated with bacteria, and despite repeated treatments with antibiotics, the primary colonies were also contaminated and therefore discarded. Two primary cultures developed from primary and metastatic tumor specimens of patients previously treated with chemotherapy underwent profound cell death after 1–2 days in culture and did not show any viable cells during the next week of observation. The other nine specimens contained a subpopulation of fast-adherent cells (FA) to the type I collagen, which initially proliferated in serum-free stem cell medium and induced floating spheroids, which are characteristic of CICs, as well as loose multicellular aggregates in 3D cultures. However, 6 of 9 primary cultures lost their clonogenic and sphere-forming capacities after several passages, which is in line with numerous observations that primary cancer cells have a finite (about 5–6 passages) lifespan [31]. In contrast, tumor cells isolated from the liver metastasis of the male patient with stage 4 colon cancer continue long-term *in vivo* and *in vitro* growth (15 passages, currently) and represent an established, CIC-enriched colon cancer cell line, CR4. Three other primary cultures are currently undergoing evolution similar to the CR4 cell line. These cells are in the process of functional, genomic, cellular and molecular characterization.

### Cellular and clonal heterogeneity of CR4 tumor cells

**Figure 1:**
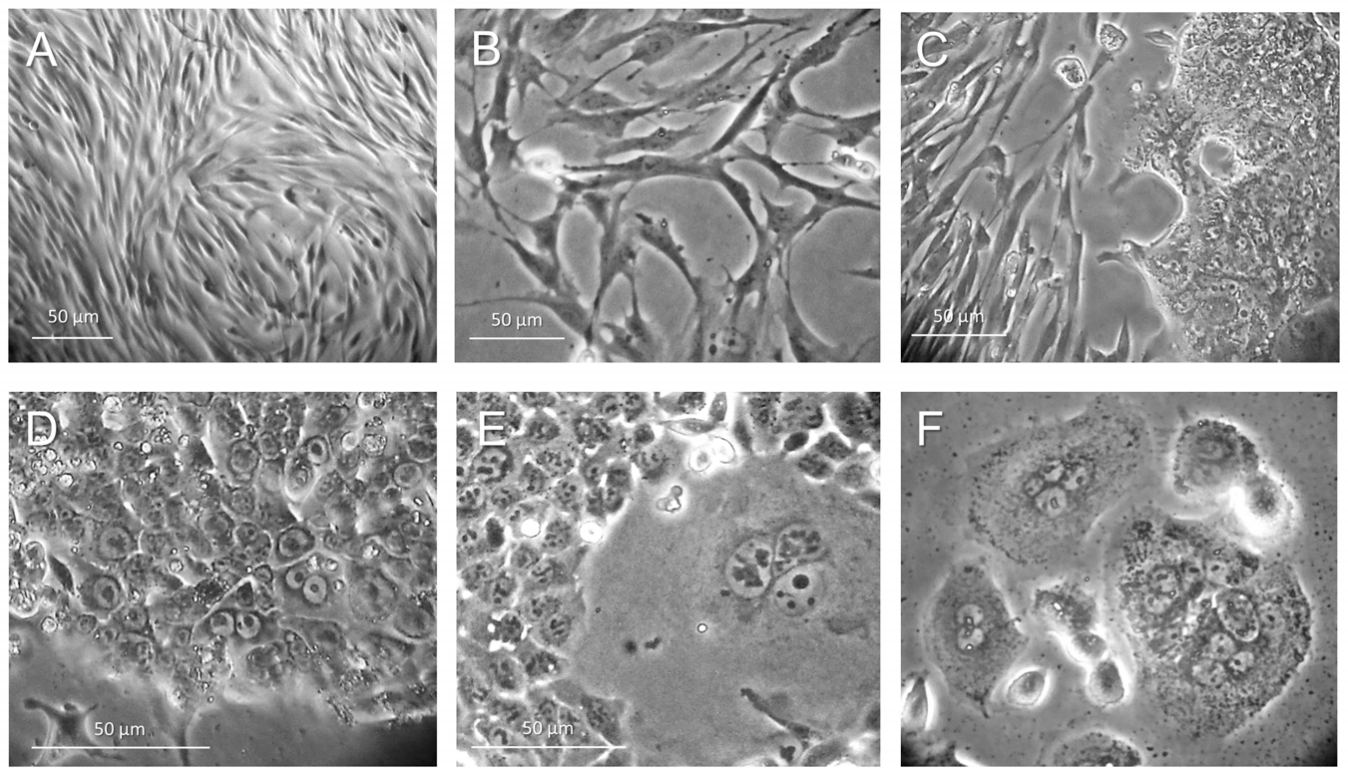
Cellular heterogeneity of the CR4 tumor cells. (**A**) Initially dominating population of the densely packed elongated fibroblast-like cells. (**B**) Sparser elongated tumor cells with processes. (**C**) Appearance of small clusters of very small cells (∼7 µm) adjacent to fibroblast-like cells. (**D**) Typical holoclone induced by small CR4 cells. (**E**) Giant multinucleated cell within CR4 holoclone of small cells. (**F**) Colony of multinucleated cells.

After initial isolation of the fast adherent to type I collagen primary tumor cells and their propagation *in vitro* in MSCB or SPC media, several different types of cell shape and clone morphology were evident: (i) densely packed long spindle-like cells representing carcinoma associated fibroblasts (CAFs) and normal fibroblasts, which were dominant cell phenotype soon after isolation and during several early passages of culturing (Figure 1A); (ii) sparse large elongated cells with dychotomized processes (Figure 1B); (iii) rare small clones containing small round cells about 7–10 µm in diameter with a very thin rim of the cytoplasm and large elongated nuclei (Figure 1C, D); and among them, (iv) rare, very large (≥100–200 µm), often multinucleated cells (MNCs; Figure 1E). We often observe such cells in both established and primary colon and prostate cancer cell lines grown under stemness-promoting conditions. These multinucleated giant cells were capable of both slow proliferation, producing either additional MNCs (Figure 1F) or large mononuclear cells.

**Figure 2:**
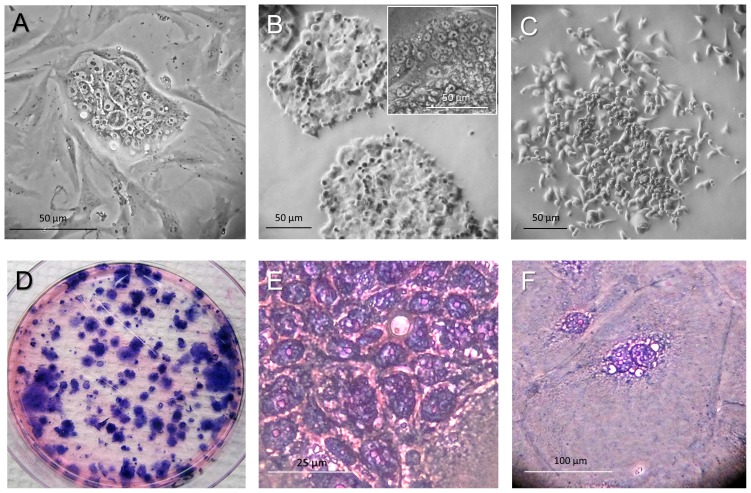
Subcloning of the small CR4 cells. (**A**) Clone of small CR4 cells surrounded by long fibroblast-like cells with dychotomized processes. (**B**) After subcloning, small CR4 cells seeded at low density in serum-free SPCM on type I collagen produced typical densely packed holoclones characteristic for stem cells of different origin. (**C**) Paraclone of elongated larger cells adjacent to small CR4 cells. (**D**) Low number of the purified small cells produced predominantly round-edged holoclones and rare paraclones adherent to type I collagen with high efficiency. (**E**) Higher magnification of the holoclone cells with large nuclei and thin rim of cytoplasm. (**F**) Large MNCs (≥200 µm; hematoxylin and eosin staining) are often located at the periphery of the holoclones.

We have continued culture of the mixed cell populations under defined stemness-promoting conditions expecting that this approach, similarly to our previously established primary prostate CIC-enriched cell line, PPT2 [32] will lead to the propagation of the rare CICs. In line with our observations, it was shown earlier that co-culture of the colon cancer cells with carcinoma-associated fibroblasts led to anincrease in the number of CICs via activation of the β-catenin pathway [33]. Finally, limited passaging of primary cells in a stem cell media led to the appearance of numerous sparse, small cell-containing, densely packed clones with smooth edges (holoclones) surrounded by elongated cells (Figure 2A). This pattern is characteristic for embryonic, induced pluripotent and other stem cell types co-cultured with fibroblasts [32], [34]. Sucloning of such holoclones led to the establishment of the purified colon cancer cell line, CR4, with all the basic features of CICs, including high tumor-initiating, 3D spheroid-and holoclone-forming capacities and expression of the pluripotency and stem cell-relevant markers (described below). Along with the putative smooth-edged holoclones, CR4 cells can induce paraclones with diffuse edges (Figure 2C), which are characteristic for progenitor cells. During the earlier passages, almost all CR4 holoclones contained uniformly packed small cells from the center to periphery of the clone (Figure 2B). However, later passages had much higher ratios of the large multinucleated cells located predominantly at the periphery of the clone, whereas small cells occupied the more central part of the clone (Figure 2 E, F). The ultra-low passages of the CR4 cells are kept in aliquotes in liquid nitrogen; currently, this line is at passage 14. For the described characterization, the frozen aliquotes were propagated either as NOD/SCID mice tumor xenografts, floating 3D spheroids or type I collagen-adherent cultures.

### Functional characterization of CR4 cells

**Figure 3:**
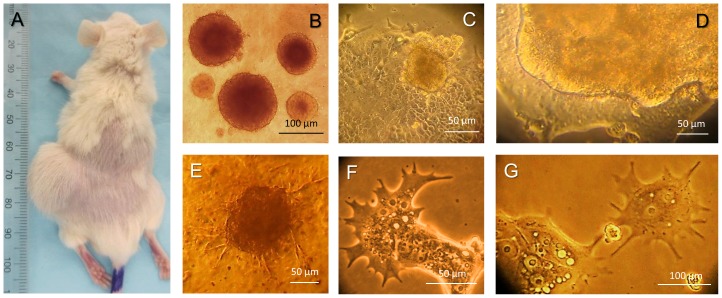
Functional characterization of the CR4 cells. (**A**) Large subcutaneous tumor induced by transplantation of the CR4 cells (1×10^3^) in NOD/SCID mouse (passage 2). (**B**) Dense floating spheroids induced by serial passaging of the CR4 cells in 3D culture on the ultra-low-adherent plates. (**C**) Three-dimensional spheroid induced on the surface of the adherent CR4 holoclone. (**D**) Formation of a large 3D organoid on the surface of the adherent CR4 holoclone. (**E**) Appearance of long processes in spheroid cells induced by CR4 cells in 3D culture containing 15% of collagen gel, which indicates their high invasive potential [35]. (**F**) Long dychotomized processes developed by adherent small CR4 cells. (**G**) Long processes of adherent MNCs.

To confirm the stemness state of the CR4 cell line, several functional assays were carried out including evaluation of the tumor-initiating potential (ability to form subcutaneous tumors in immunodeficient NOD/SCID mice), sphere-forming capacity (ability to form dense floating spheroids in nonadherent 3D cultures) and clonogenicity (ability to form adherent to type I collagen holoclones). We have determined that the CR4 cells possess high efficiency in induction of tumors in NOD/SCID mice (Figure 3A) after serial subcutaneous transplantations of the relatively low cell number. Thus, 1×10^3^ CR4 cells or 2–3 floating spheroids induced large tumors in all mice (6 mice per each group). To establish clonogenic and sphere-forming capacities of the CR4 cells, a known number of cells were seeded on type I collagen-coated or ultra-low adherent plates, respectively. After one week of incubation, induced adherent clones or floating spheroids were counted and the clonogenic efficiency was calculated as the ratio of the number of seeded cells compared to the number of induced colonies or spheroids. In particular, we determined that after seeding unsorted CR4 cells of passage 13 in serial dilution (250, 100, 50 and 25 cells/well of 96-well plates) about one cell in 10 (10%) induced perfect holoclones (Figure 2B, D). Sphere-forming efficiency of the CD133-positive fraction of the CR4 cells in 3D culture with 5% type I collagen in SPCM was higher compared to the CD133-depleted cell populations (8.4±2.6 versus 3.7±0.6 spheroids per 100 seeded cells/well, respectively). Both holoclones and spheroids were packed with very small cells expressing high levels of common stemness markers, including EpCAM, CD133, CD44, CD166 and Lgr5 (described below). Although the CD133 (clone 293C3), as well as any other common markers, is not ideal for colon CICs, nevertheless it allows for additional enrichment of the tumor-initiating and sphere-forming fraction of the CR4 cells.

The aggressive nature and exceptional sphere-forming/clonogenic capacity of CR4 cells were demonstrated by their ability to produce floating spheroids not only in non-adherent cultures (Figure 3B), but also by budding of the adherent to type I collagen holoclones and consequent detachment of the formed spheroids (Figure 3C). The CR4 holoclones were also able to produce the large adherent multilayer organoids directly above the holoclone surface (Figure 3D). Floating CR4 spheroids behaved differently in 3D culture systems with and without type I collagen gels in stem cell media (MSCBM or SPCM). Thus, spheroids grown with no or low percent of collagen gel (up to 5%) usually have smooth edges (Figure 3B), whereas higher gel concentration (up to 10–15%) led to the appearance of multiple processes, which formed asterisk-like structures surrounding spheroids (Figure 3E). Such phenotype of floating spheroids was recently associated with high metastatic capacity of particular cancer cells [35], which is in line with the fact that the CR4 cell line was established from liver metastasis of the colon cancer patient. Similarly to 3D culture, the invasive nature of the CR4 cells was reflected by their ability to develop long, large, dichotomized processes on the outer edge of the adherent clones (Figure 3F). The large multinucleated cells on the periphery of the clones, described above, as well as single gigantic multinucleated cells, also often displayed long and dychotomized processes (Figure 3G).

### Phenotypic profiling of CR4 cells

**Figure 4:**
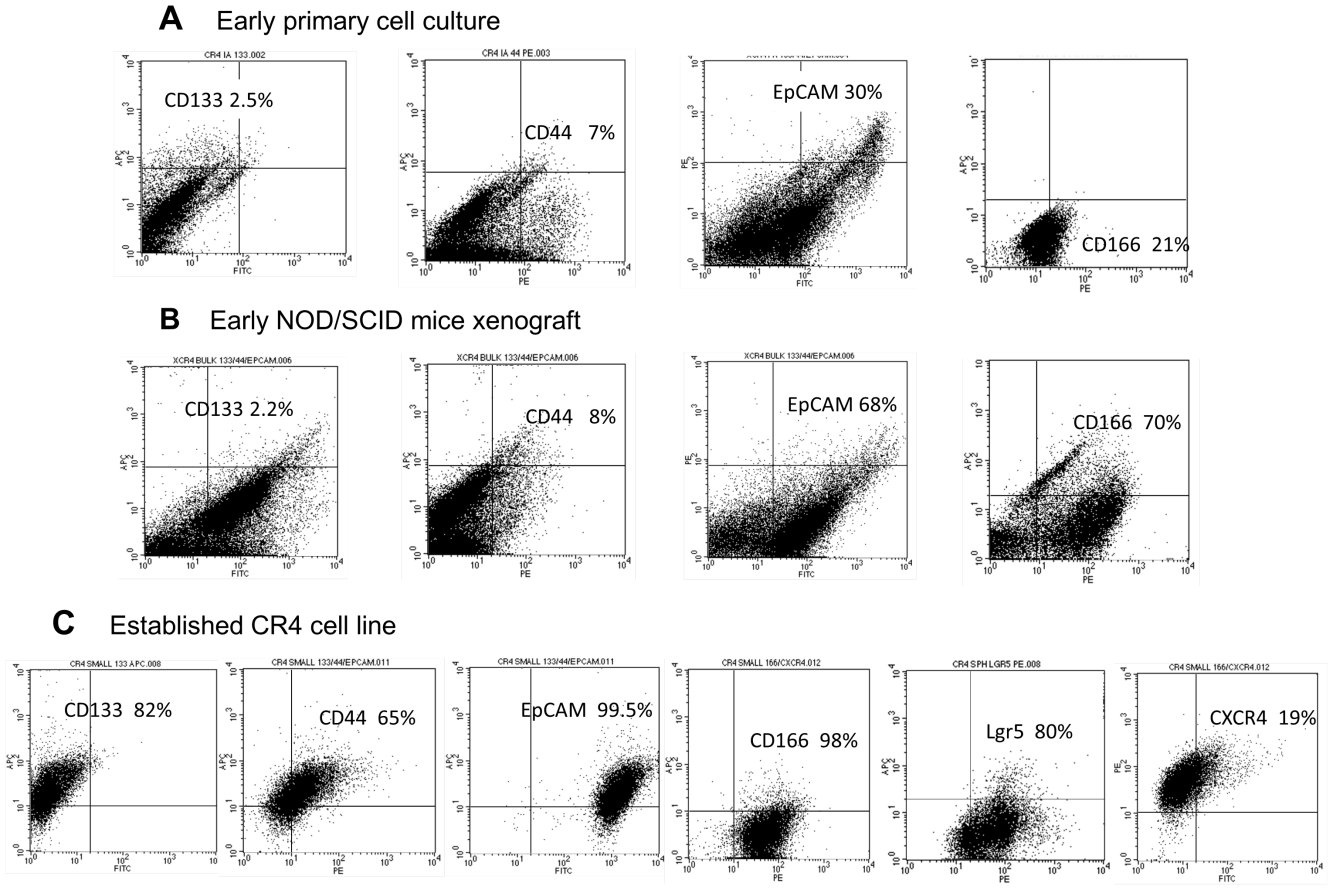
Molecular characterization of the CR4 cell line: expression of cell surface markers. (**A**) Representative FACS analyses of the different cell surface markers expression in early primary culture of the fast-adherent CR4 cells grown on type I collagen in MSCB medium. (**B**) FACS analysis of the first passage of FA tumor cells isolated from NOD/SCID mice tumor xenografts induced by early-passage CR4 cells. (**C**) Dramatic increase in the expression of the common markers of stemness, including CD133, CD44, CD166, Lgr5 and EpCAM in the late-passage (p13) CR4 cells. Note that 19% of cells also expressed marker of CICs with metastatic activity, CXCR4, which was identified in multiple human cancers.

**Figure 5:**
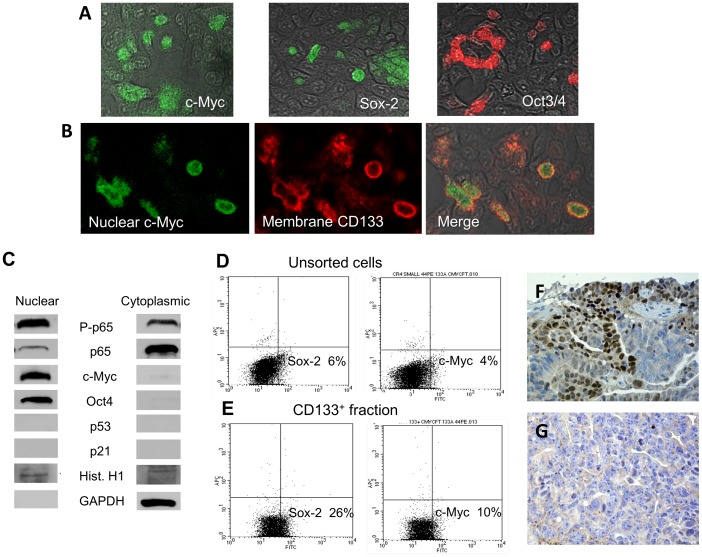
Expression of key pluripotency markers in CR4 cells. (**A**) Nuclear localization of key pluripotency markers, c-Myc, Sox-2 and Oct3/4. (**B**) Co­localization of nuclear c-Myc with high membrane expression of CD133. (A, B: Immunocytochemical analysis of CR4 cells grown on type I collagen-coated chambered slides). (**C**) Western blot analysis confirms expression of c-Myc and Oct-3/4 in nuclear fractions of the CR4 cells. In contrast, they are negative for p53 and p21. Nuclear fraction also expressed higher levels of the phosphorilated p65 (which indicate constitutive activation of NF-kB), whereas unphosphorilated p65 was located predominantly in a cytoplasm. (**D, E**) Representative FACS analyses show higher expression of Sox-2 and c-Myc in CD133-positive cells compared to unsorted cells. (**F**) Immunohistochemical analysis shows high nuclear expression of Sox-2 in the CR4-induced NOD/SCID mice tumor xenograft. (**G**) Negative control (tissue section without incubation with primary anti-Sox-2 Abs).

As we mentioned above, subcloning of the small-cell-containing holoclones led to dramatic enrichment of cells expressing high levels of stemness and pluripotency markers (Table 1; Figures 4 and 5). In general, stem cell phenotype *in vitro* is dynamic due to the dual nature of CICs (i.e., their ability to self-renew and to generate committed progenitors). Thus, early and intermediate passages of primary CR4 cells had unstable phenotype expressing highly variable levels of CD133, CD44 and EpCAM (Table 1 and Figure 4A). The NOD/SCID mice tumor xenografts induced by these cells expressed similar levels of all studied markers (Figure 4B). In contrast, purified CR4 cells retain relatively stable phenotype in 3D and adherent to the type I collagen cultures. Thus, three independent FACS analyses during the last 7 months (passages 6–14) have shown that virtually the entire population of CR4 cells remains undifferentiated (only 3–5% expressed marker of differentiation, pan-keratin), and the majority of cells express high levels of CD133 (62–82%), CD44 (65–99%), CD166 (97–98%), EpCAM (98–99%) and Lgr5 (80–83%; representative FACS data are shown on Figure 4C). In particular, about 65% of cells coexpressed high levels of CD133 and CD44. Importantly, about 20% of CR4 cells are positive for marker of metastatic activity, CXCR4.

**Table 1:** Phenotypic evolution of the CR4 tumor cells (FACS analysis).

	Early Primary cultures (% of positive cells)		Intermediate (% of positive cells)	Established CR4 (% of positive cells)
Marker	Adherent*	1^st^ xenograft**	Adherent	Adherent***
**EpCAM**	3.4±1.6	65±3	84±5	97±2.7
**CD133**	1.5±1	2.2±0.6	15±5.5	62±9.7
**CD44**	7.5±0.5	7±1	35±13	82±17.3
**CD166**			70±5	97±1
**CXCR4**	1±0.5	1.2±04	20±2.2	82±17.3
**Lgr5**			81.5±0.5	83±2
**CXCR4**			10.5±1	19±2
**Sox-2**			3±0.5	20±1.5
**c-Myc**			1±0.2	10±2.8

* Early passages (# 2–4) of the fast-adherent to type I collagen CR4 cells isolated from patient tumor.

** Fast-adherent cells isolated from the first passage of NOD/SCID mice tumor induced by early passage.

CR4 cells.

*** Late passage of CR4 cells, which were twice transplanted to NOD/SCID mice and grown under.

stemness-promoting conditions (passages 11–13).

*Note:* Data from at least three different FACS analyses were expressed as means ± SD for each marker.

Initially very low cell number didn't allow more complete evaluation of the stemness/pluripotency markers.

**Figure 6:**
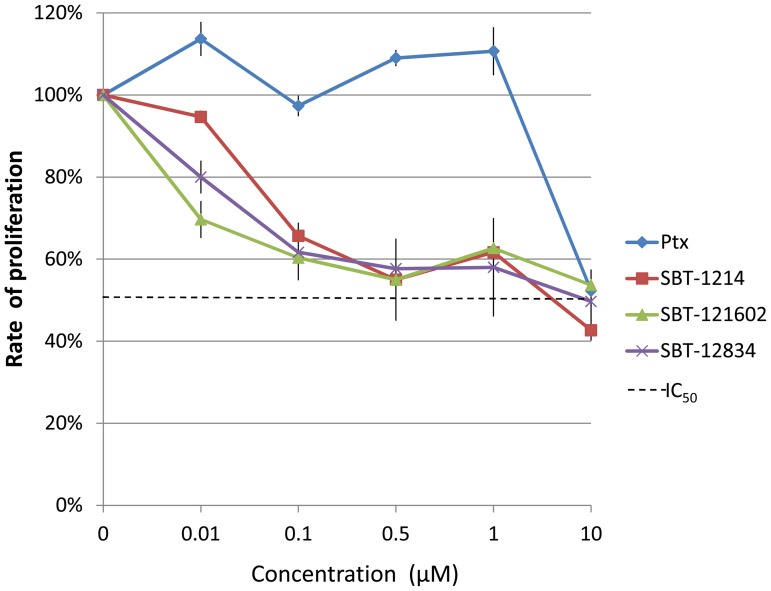
CR4 cells are highly resistant to treatment with cytotoxic drugs (comparative cytotoxicity of paclitaxel and new-generation taxoids against CR4 cells). Commonly used Paclitaxel (Taxol) in doses lower than 10 µM is not effective against CR4 cells and often increases their proliferation. In contrast, several new-generation taxoids induce dose-dependent inhibition of proliferation of these potent tumor-initiating cells. (MTT assay after drug treatment for 48 hr). The obtained *p* values for all the drugs and all the drug concentrations were much smaller that 0.05. The largest *p* value was obtained for SBT-1214 at 10 nM concentration (*p* = 0.0131); in particular, at 10 µM concentration of SBT-1214 *p* = 0.00032.

Using immunocytochemical, FACS and western blot analyses, we have shown that a significant ratio of the CR4 cells express several key markers of pluripotency, including Sox-2, Oct3/4 and c-Myc. Nuclear localization of these markers shown by ICC (Figure 5A, B) was confirmed by western blotting (C), which has demonstrated their expression in the nuclear protein fraction and their absence in the cytoplasmic one. We have found that the CD133^+^ fraction of the CR4 cells express higher ratios of several markers of pluripotency compared to the unsorted CR4 cells. Thus, FACS analysis has shown that more than a quarter of the CD133^+^ population expressed Sox-2 and 10% of the population were positive for c-Myc (Figure 5D). In contrast, unsorted CR4 cells and CD133-negative fraction expressed lower levels of these pluripotency markers (6 and 4%, and 1.4 and 0.8%, respectively; Figure 5E; negative fraction is not shown). Colocalization analysis has shown that only cells with the highest expression of CD133 usually have nuclear staining for the pluripotancy markers (Figure 5B; only c-Myc is shown). High proportion of the Sox-2­positive cells and its nuclear localization was also confirmed by IHC of the NOD/SCID mice tumor xenografts (Figure 5F). [Of note, the use of the anti-Oct3/4 (Santa Cruz Biotechnology, CA, USA) for FACS analysis provided suspiciously high ratios of positive cells (more than 90%) and these data were not included.] Importantly, the CR4 cells, similarly to the prostate PPT2 CIC-enriched cell line [32], did not express the two major regulators of apoptosis and tumor suppressor genes, p53 and p21 (Figure 5C). In addition, the nuclear fraction of the CR4 cells grown on type I collagen in stem cell medium as separate holoclones strongly expressed phosphorylated p65, which means that NFkB is constitutively overexpressed in colorectal CICs. In contrast, unphosphorylated p65 was located predominantly in the cytoplasmic fraction. All of the above may partially explain the high tumorigenic and clonogenic capacities and exceptional drug resistance of this CIC-enriched cell line. In particular, CR4 cells are highly tolerant to treatment with commonly used cytotoxic drugs such as Paclitaxel or Taxol (Figure 6). Thus, after 72-hour treatment in concentration range from 10 nM up to 10 µM (MTT assay), CR4 cells have shown little or no cytotoxicity; moreover, they often increased their proliferation in response to lower doses of the paclitaxel. We have found that several members of new-generation taxoids, including SBT-1214, SBT-121602 and SBT-12834 are more effective against CR4 tumor-initiating cells. The IC50 death rate was reached at ≥10 µM of all drugs concentration. Of note, the promising efficacy of low concentrations of new-generation taxoids can be further improved by their combination with the synthetic derivative of curcumin, CMC2.24 (unpublished data), which is line with our previous study [32].

### Histopathological and IHC analyses of the NOD/SCID mice tumor xenografts

**Figure 7:**
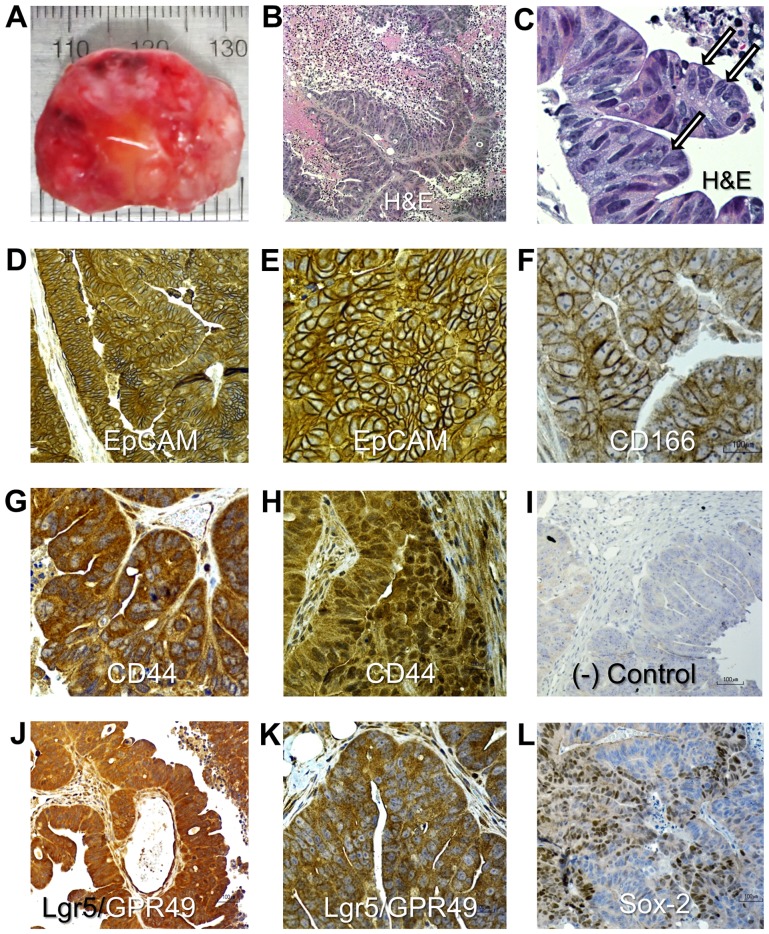
Histopathological and immunohistocheminal analyses of the CR4-induced mice tumor xenograft tissues. (**A**) Large tumor induced by transplantation of 1×10^3^ CR4 cells. (**B**) Hematoxylin ­and eosin-stained tissue section shows classic histologic features of human metastatic colon cancer. (**C**) High power magnification of region shown in (**B**); arrows show giant multinucleated cells within the papillary structures. (**D, E**) Strong cell surface expression of the epithelial marker, EpCAM. (**F**) Strong cell surface expression of CD166. (**G**) Cytoplasmic expression of CD44. (**H**) Microscopic focus with strong nuclear and weaker cytoplasmic expression of CD44. (**I**) Negative control (primary Abs were omitted). (**J, K**) Strong and moderate expression, respectively, of the colon stem cell marker, Lgr5/GPR49. (**L**) Strong nuclear expression of the pluripotency marker, Sox-2 in large areas of the tumor.

As we mentioned above, subcutaneous transplantation of a relatively low number of CR4 cells (1×10^3^ of the dissociated CR4 cells or 2–3 floating spheroids) induced large vascularized tumors in all injected NOD/SCID mice (Figures 3A and 7A). The hematoxylin-and eosin-stained tissue sections of the mice tumor xenografts showed classic histologic features of human metastatic colon cancer (Figure 7B, C). Highly atypical epithelial cells formed villous-like structures with prominent elongated nuclei and, consistent with poorly differentiated adenocarcinoma, numerous atypical mitotic figures. Central necrosis was usually present. Numerous large multinucleated cells were evident at higher magnification (Figure 7C; arrows). Immunohistochemical analysis revealed that entire tumor area (but not tumor stroma) expressed high levels of the membrane-localized EpCAM (Figure 7D, E). Similar patterns and levels of expression were characteristic for CD166 (F). In contrast, immunostaining with polyclonal CD44 (clone F10-44-2; Invitrogen/Biosources, USA) revealed three different patterns of expression in different areas of the same tumor, i.e. clearly membrane (Figure 7G), as well as cytoplasmic and clearly nuclear in other parts (Figure 7H). High cytolasmic expression of the common marker of colon stem cells and CICs, Lgr5, was evident in some tumor parts (Figure 7J), whereas in other parts, it was either moderately or weakly expressed (K), or even absent. Large areas of the tumor xenografts expressed strong nuclear staining for the pluripotency marker Sox-2 (Figure 7L).

### RNA-Seq complete transcriptome profiling of CR4 small versus bulk tumor cells

Using RNA-Seq, we performed a functional genomic analysis in tumor-initiating fractions of CR4 (small) cells grown adherent to type I collagen versus grown as 3D spheroids, in comparison to the bulk tumor cells (long and dychotomized cells grown under standard culture conditions). Using a HiSeq 2000, we sequenced between 40 and 50 million reads per biological sample, as previously described [35], [36], [37]. Briefly, libraries for NGS were made from 100 nanograms of total RNA. The RNA samples quality was assessed with an Agilent Bioanalyzer. All RNA samples had a RIN above 8. Sequencing libraries were created using the Illumina TruSeq Stranded mRNA LT kit according to manufacturer recommendations. The quality of each library was evaluated with the Agilent bioanalyzer high sensitivity assay, and quantified by qPCR (Kappa Biosystem, CT). The libraries were pooled together at 10 nM based on the qPCR results, and then the pool was quantified again by qPCR. The pooled library was sequenced in one lane of a HiSeq2000 paired end 100bp flow cell. This allowed us to detect which transcripts are differentially expressed among these types of CR4 cells, thus building a comprehensive picture of the activated or repressed signaling pathways. We have found that small CR4 cells grown either as separate holoclones adherent to type I collagen or as 3D floating spheroids possess a large number of differentially expressed genes in comparison to the bulk tumor cells grown under standard culture conditions. Thus, in adherent small CR4 cells and 3D spheroids, 357 and 365 genes, respectively, were overexpressed compared to the CR4 long (bulk) tumor cells. Among these genes, 287 were commonly upregulated, which means that both culture conditions allow for maintenance of the CR4 small cells at stemness state. In particular, both small adherent and 3D spheroids expressed up to several orders of magnitude of upregulation in the following: (i) multiple common markers of stemness, including CD44, CD24, EpCAM, ESA, Lgr5, ALDH1A1 and others; (ii) growth factors, including the epidermal (EGF), fibroblast (FGF) and transforming growth factor-beta (TGFβ) family members; (iii) transcription factors (TFs), including multiple homeobox (CDX1, CDX2, CEACAM6, MSX2) and pluripotency TFs (POU5F1B, Oct4, Sox-2, Sox-9; (iv) inflammatory cytokines and their receptors, including IL18, IL20, IL2RG, IL20RA and others; (v) ABC transporters; (vi) genes responsible for transfer of high energy phosphate from mitochondria (CKMT1 and CKMT1B) and the members of cytochrom P450 family, including CYP2B6, CYP2J2, CYP2S1 and others. Of interest, the tumor-initiating fraction of the CR4 cells overexpressed multiple genes controlling cell-to-cell adhesion, including cadherins (CDH 1, 3 and 17, and CDHR5); intergins (ITGB4); genes encoding tight-junction proteins (CLDN3, 4 and 6, COL17A1 and others); and keratins (KRT19, 20, 23 and KRTAP3-1). This finding supports our traditionally used approach for initial enrichment of the prostate and colon tumor-initiating cells based on their ability to adhere to type I collagen-coated surfaces within 15–20 minutes of incubation. The obtained raw RNA-Seq data were submitted to the National Center for Biotechnology Information (NCBI) Gene Expression Omnibus (GEO), a public functional genomics data repository (the ID number is *GSE56660*).

### STR profiling of the original tumor and isolated CR4 cells

Both the parental tumor tissue and the isolated tumor-initiating population of this tumor (xenograft CR4 cells) were subjected to the short tandem repeat (STR) profiling by the GRCF at John Hopkins DNA Services/FAF. STR profiling by the GRCF is carried out following the ANSI/ATCC ASN-0002-2011, Authentication of Human Cell Lines: Standardization of STR Profiling. According to this report, both samples represented unique human profiles, which do not match any profile in either ATCC (American Tissue Culture Collection) or JCRB (Japanese Collection of Research Bioresources). The STR profiles of the parental tumor and the established cell line after xenografting in NOD/SCID mice are identical with the exception of the loss of the Y-chromosome in CR4 cells, which is a common phenomenon for different types of human malignancies. Thus, the Y-chromosome loss was reported in 20 out of 26 karyograms of CIC-enriched highly tumorigenic pancreatic cell line [34].

## Discussion

It is largely accepted now that effective anticancer therapies should be focused not only on the bulk mass of the tumor, but most importantly, on functionally significant cells possessing high tumor-initiating capacity and high resistance to treatment, i.e. CICs. It is also well established that human tumors are highly heterogeneous both intra-and interindividually. Given the fact that the majority of established long-term passaged cancer cell lines do not reflect either complexity, or individual features of the parental tumors [3], [38], it is conceivable that modern cancer research and anti-cancer drug development should be based on novel *in vitro* and *in vivo* systems allowing for physiologically and clinically more relevant modeling and more objective evaluation of drug efficacy. Molecular, biochemical, genomic and proteomic characterization of human tumors aims to discover novel targets that potentially can cure cancer. But in reality, clinically and physiologically relevant targets are covered by noise from the dominating cell populations in unselected, bulk tumor masses, often contaminated with different levels of non-tumor cells, including normal fibroblasts and CAFs, endothelial cells, pericytes, inflammatory and other cells. Therefore, the selection of clinically and physiologically appropriate molecular targets should be fundamentally based on the selection of appropriate, clinically and physiologically significant cells, even if they represent a very minor population within the individual tumor. Secondly, profound intra-and inter-individual variations in grade of differentiation, morphology, level and type of chromosomal abnormalities, as well as the ratio of tumor-and metastasis-initiating cells require the availability of a large spectrum of patient-derived low-passage cell lines. Moreover, such primary cell lines should preferably be established from multiple tumor foci in order to better reflect intratumoral complexity, heterogeneity and hierarchy. As we mentioned above, the establishment of patient-derived low-passage cancer cell lines from fresh tumors *in vitro* remains technically difficult [18]–[20], [27], [31], [39], [40]. In part, this is due to the natural heterogeneity and multiclonality of human cancers, which makes it difficult to isolate the most clinically significant malignant clone(s) given the limited size and number of fresh tumor specimens. In addition, the frequent incompatibility of research needs (availability of fresh, promptly microdissected malignant specimens) and routine clinical processing of resected tumors (usually delayed pathological evaluation and their preferential storage in fixatives) further complicates this situation. Even more difficult is the establishment of purified CIC lines due to the rarity of these cells within the tumor and their often unpredictable behavior *in vitro*. However, although the commercially available established cancer cell lines are very convenient, user-and budget-friendly, and have for many decades remained to be a major resource for cancer research and anti-cancer drug development, several serious concerns were recently raised. While established cancer cell lines do contain different ratios of clonogenic and tumor-initiating cells, the long-term maintenance of these lines inevitably leads to dramatic deviation of their features from those of parental tumors. Thus, a recent NIH-based study has demonstrated that the most commonly used collection of the established cancer cell lines, NCI-60, has no correlation with the original clinical specimens [3]. Here we report the establishment of the colon CR4 cell line, which is highly enriched with tumor-initiating and holoclones-and 3D spheroid-forming cells with stem cell properties. Under described conditions, the vast majority of the fast adherent to type I collagen tumor cells initially grew in both 3D and adherent culture systems (in contrast to a previous study [27] that found that only 3 of 31 tumor specimens grew initially*in vitro*). However, these cells usually lost their clonogenic capacity after several passages. In contrast to all previously studied commercial cell lines, but similarly to our recently established purified prostate cancer stem cell line, PPT2 [32], CR4 cells produced perfect floating spheroids not only in 3D culture, but also on the surface of the type I collagen-adherent holoclones, which reflect their exceptional clonogenic/sphere-forming potential. There is a large body of evidence that 3D tumor cell cultures more accurately reflect the complex *in vivo* microenvironment than the commonly used two-dimensional cell monolayers [35], [41]. It was also shown that human colorectal cancer cells grown on type I collagen in serum-free medium undergo an epithelial-mesenchymal-like transition [42]. On the other hand, EMT was shown to generate stem-like cells [43]. In particular, collagen type I inhibited cell differentiation, increased clonogenicity and promoted expression of CD133 and Bmi1, indicating that it promoted expression of a stem cell-like phenotype in colon cancer cells [42]. In this context, culturing the adherent to a type I collagen CIC-enriched cell population in a serum-free stem cell medium can provide a useful tool for enrichment and maintenance of the tumor-initiating fraction of cancer cells. Although the morphological manifestation of CICs has not been thoroughly investigated, partly because of the rarity of these cells and a lack of highly specific markers, accumulating data demonstrate that giant multinucleated cells, which are present in relatively low numbers in malignant tissues and cell lines, and can be enriched after treatment with standard anti-cancer drugs [44], [45], possibly represent a subpopulation of cancer stem cells [46].

Recently, the establishment of a highly tumorigenic CIC-enriched pancreatic cancer cell line, JoPaca-1, has been reported [34]. Authors have found a significant decrease in the ratio of cells expressing CD133, ALDH and co-expressing CD44/CD24/ESA after 13 consequent passages (80% of the CD133^+^, 47% of CD44/CD24/ESA-positive cells and 28% of ALDH^+^ cells at passage 6 versus 34%, 3% and 10% at passage 19, respectively). In contrast to JoPaca-1, the ratio of the CR4 cells expressing stemness markers was significantly increased followed by passaging under defined stemness promoting conditions. Since this phenotype remained stable during the last 7 months, we can suggest that CR4 cells represent purified immature CICs, which are predominantly self-renewing under described conditions. As we have shown, the majority of the established CR4 cells, as well as a significant proportion of cells from NOD/SCID mice tumor xenografts induced by these cells, express high levels of CD133, CD44, CD166, EpCAM and Lgr5. We observed widespread membrane, cytoplasmic and nuclear expression of CD44 after immunostaining with polyclonal Abs, which may reflect complex functional roles of this marker. Thus, it is implicated in a wide variety of physiological and pathological processes as a cell surface adhesion molecule that interacts with hyaluronic acid, the most abundant stromal component. In particular, this cell surface hyaluronan receptor may promote tumor growth and metastasis serving as an anchor for matalloproteinase MMP9 [47]. In addition, sequential proteolitic cleavages of CD44 in the ectodomain and intramembranous domain lead to the release of a CD44 intracellular domain fragment, which acts as a signal transduction molecule after translocation to the nucleus [48]. About 20% of CR4 cells are positive for marker of metastatic activity, CXCR4, which is in agreement with their origin from liver metastasis. The CXCR4, cell surface receptor for the chemokine CXCL12 (SDF-1), plays a central role in cancer cell proliferation, invasion, and dissemination in the majority of malignant diseases. Accumulating evidence suggests a key role of the CXCL12/CXCR4 axis in the metastatic niche signaling that regulate CICs [49]–[53]. In line with recent studies [54], [55], we observed Lgr5-positive clusters of cells with strong and moderate cytoplasmic staining in poorly differentiated mice tumors. In contrast to the previously reported spontaneously immortalized cancer cell lines [56], which express high levels of p53 and markers of differentiation, the CR4 line is immature and negative for p53 and p21. Complex mutual regulation within the p53/p21 axis and p53-regulated miRNAs contribute to tumor suppression by controlling the expression of such central processes as cell cycle progression, EMT, stemness, metabolism, cell survival and angiogenesis; therefore, aberrations in this network play a critical role in tumor development [57]. The CR4 cells possess high nuclear expression of the phosphorilated p65, which means constitutive activation of the NF-κB in these cell lines. It was previously shown that in colorectal and gastric cancers, inhibition of nuclear translocation of p65 is associated with reduced cancer cell proliferation, induction of apoptosis and tumor shrinkage [58], [59]. NF-κB p65 protein was shown to be expressed in a small subpopulation of CD133^+^ prostate cancer cells and overexpressed in 80–100% of metastatic lesions.

In addition to FACS, ICC, IHC and western blot analyses, using RNA-Seq, we obtained precise measurement of levels of transcripts, which are differentially expressed in the tumorigenic fraction of CR4 cells in comparison to bulk (non-stem) tumor cells from the same tumor (about 360 genes). Next-generation sequencing (NGS) provides a unique opportunity to uncover the genomic status of functionally different cell types. In general, RNA-Seq provides a far more precise measurement of levels of transcripts and their isoforms than other methods. Functional characteristics of many of the differentially expressed genes, including common markers of stemness and key pluripotency genes, multiple transcription and growth factors involved in cancer development and progression, overactivated ABC transporters and others, clearly confirm the stemness state and high drug resistance of the CR4 cell line. Of interest, the tumor-initiating fraction of the CR4 cells overexpressed multiple genes controlling cell-to-cell adhesion, including cadherins, intergins and tight-junction proteins. This finding supports our traditionally used approach for initial enrichment of the tumor-initiating cells based on their ability to adhere to type I collagen-coated surfaces within 15 minutes of incubation. The STR profiling of CR4 tumor xenografts revealed that, similarly to our recently established prostate CIC cell line, PPT2 [32], CR4 cells possess the loss of the Y-chromosome, which is in line with observation that deletion of the Y-chromosome is associated with the simultaneous inactivation of tumor suppressor genes [60], [61]. In general, the loss of the Y-chromosome and numerical abnormalities of the X-chromosome are frequent phenomena observed in different cancer types [62], [63] and most tumors possess at least one form of genomic instability [64]. The absence of p53 and p21 in CR4 cell line reflects their extremely high resistance to drug treatment. The p53 transcription factor regulates multiple biological functions via regulation of the expression of a wide variety of genes involved in apoptosis, growth arrest, inhibition of cell cycle progression, differentiation and accelerated DNA repair.

There has been considerable debate as to whether there are specific CIC markers. In particular, colorectal CICs have been initially identified using CD44 or CD133 either alone [7]–[9], or in combination with other markers, such as EpCAM, CD166, CD29, CD24, LGR5 and aldehyde dehydrogenase 1, ALDH1 [65]–[67]. Accumulating data indicate that tumor-initiating cells in many cancer types cannot be demarcated solely by the expression of common cell surface markers [68]–[70]. Although both CD44 and CD133 were reported as putative markers for many cancer-specific CICs, including colorectal cancer, it is still unclear whether they are of equal functional importance. It was shown that CD133^+^ normal stem cells at the base of the adult intestinal crypts (a stem cell niche) not only generate the entire intestinal epithelium, but give rise to all the neoplastic cells in mice colon tumors [71]. The proportion of CD133^+^ cells in colon cancer metastases is higher than in primary tumors [72], which reflect the well-known fact that metastatic lesions are more resistant to treatment. However, another study has shown that only a knockdown of CD44, but not CD133, strongly prevented clonal formation and inhibited tumorigenicity in the mice xenograft model [73], and that CD44^+^ is not colocalized with CD133^+^ cells within colorectal cancer. Similar results reported by Horst and colleagues showed that the expression of CD133 correlates with that of CD166, while both do not correlate with CD44 [74]. However, this data contradicts multiple reports which not only show colocalization of the CD133 and CD44 in several types of human cancer [7], [44], [68], [75]–[77], but also suggest their combined expression as the best CIC marker [68], [71]. Such inconsistency may be due to the high heterogeneity of clinical specimens, the comparison of the data obtained on clinical specimens with data obtained on established cell lines, the diversity of experimental approaches, and the lack of highly specific CIC markers. A recent study has demonstrated that, in contrast to the established cancer cell lines, CD133^+^ cells in primary colorectal cancer samples showed a unique genomic aberration profile [20], which additionally highlights the point that the use of established cancer cell line is questionable [3], [20], [78].

In conclusion, our data indicate that the CR4 cells isolated from liver metastasis of colon cancer patient represent an established cell line, which possesses classical features of CICs, including high tumor-initiating, clonogenic and sphere-forming capacities and exceptional resistance to anti-cancer drugs. All of the above demonstrates that these cells are a highly valuable tool for CIC research and anti-cancer drug development.

## Methods

### Tumor Tissue Specimens, Isolation of Cancer Cells and Primary Cultures

#### Ethics Statement

Fresh CR tumor specimens were obtained from SBU patients with clnically proven CRC via a research protocol approved by Stony Brook University Committees on Research Involving Human Subjects (CORIHS; #337148-3). Informed written consent was obtained on all participants. All experiments involving the use of animals were carried out in strict accordance with the recommendations in the Guide for the Care and Use of Laboratory Animals of the National Institutes of Health, via a research protocol that was approved by Stony Brook University Institutional Animal Care and Use Committee (IACUC #: 2011-1683 - R1 -11.14.14 – MI).

Immediately following resection, tumors were placed in standard culture medium with antibiotics and antifungal, and transported to the laboratory on ice. Fresh tumors were mechanically and enzymatically disaggregated into single cell suspension at sterile conditions. Specimens were first minced into 1–2 mm^3^ chunks, rinsed with Hank's balanced salt solution and then incubated for 1 hour in humidified atmosphere containing 5% CO_2_ at 37°C in serum-free McCoy medium (GIBCO, Invitrogen, Carlsbad, CA, USA) containing 200 units/ml Collagenases type II and type IV (Sigma-Aldrich), 120 µg/ml penicillin and 100 µg/ml streptomycin. Cells were further disaggregated by pipetting and serial filtration through cell dissociation sieves (size 40 and 80 meshes; Sigma-Aldrich). Single cell suspensions were placed on type-I collagen-coated dishes (Biocoat; Becton Dickenson, Bedford, MA) in serum-free Mesenchymal Stem Cell Growth Medium (MSCGM; Lonza) or Stemline Pluripotent Culture Medium (SPCM; Sigma-Aldrich). One portion of the fast adherent (FA) cells, which were attached to type I collagen within the following 15–20 min was used for generation of floating 3D spheroids, and another one remained on the type I collagen-coated dishes for further propagation. The FA-depleted cell suspensions were resuspended in standard McCoy medium supplemented with 10% fetal bovine serum (Gibco, USA) and placed either on regular culture dishes or on the type I collagen-coated ones. The leftover of the undissociated tumor pieces was removed from the dissociation sieves and also placed in culture. The media were changed 2–3 times per week. Penicillin, streptomycin and TrypLE were obtained from Invitrogen (Grand Island, NY, USA).

### Generation of floating multucellular spheroids and *in vitro* clonogenic capacity evaluation

Four-five hunderd cells of particular phenotype were seeded on each well of the Ultra-Low Attachment (ULA) 6-well plate (Corning, Lowell, MA) in serum-free MSCB medium (Lonza) or Stemline Pluripotent Stem Cell Culture media (SPCM; Sigma) containing 5–15% of type I collagen gel and examined after 1 week of culturing under standard conditions. Fresh medium was added after one week of culturing, every third day. After initial culturing during 1–2 weeks on ULA plates, primary spheres were gently disaggregated by repeated pipetting and transferred into ULA flasks for further propagation and maintenance. Growing floating spheroids are usually enriched with tumor-initiating cells and do not contain contaminating cell types such as fibroblasts, therefore we used them for further propagation of cancer cells by serial passaging in vitro, for testing clonogenic capacity of the candidate cell phenotypes and for induction of human tumor xenografts in immunodeficient mice. For evaluation of the clonogenic/sphere-forming capacity, cells were counted with Cellometer Auto T4 (Nexcelom Bioscience LLC, MA), resuspended in 1∶4 type I collagen//SPCM or 1∶4 Matrigel/MSBM and known cell numbers were plated on ULA plates. One week after initiation plates were inspected for floating sphere growth and compact well-shaped spheroids were counted. Spheroids were serially passaged by gentle dissociation and mixing with a new type I collagen/MSCBM or type I collagen/SPCM and reseeded on ULA plates of flasks for further propagation and analysis. Sphere-forming efficiency was calculated as a ratio of seeded cells to the number of produced 3D spheroids.

### Adherent to type I collagen cultures

The CD133^high^/CD44^high^ cells (4–5×10^3^ cells/well were seeded onto the type I collagen-coated 6-well plates, and the experiments were initiated 24 h later, upon sub-confluency. The regular MSCB medium was replaced with treatment media containing SB-T-1214 at selected concentrations (0.001–0.1 µg/ml). Treatment media was removed 24 hr later, followed by washing with regular MSCBM and dissociation of cells with an Enzyme-free dissociating reagent (Chemicon International). Upon termination (48 hr from start), cell viability and cell death was analyzed through flow cytometry.

### Generation of human tumor xenografts and *in vivo* tumorigenicity tests

All experiments involving the use of animals were performed in accordance with SBU institutional animal welfare guidelines. NOD/SCID mice (Charles River Laboratories, Wilmington, MA, USA) were maintained under defined conditions at the SBU animal facility. Aliquots of the particular cell populations were counted and cell viability was determined by iconventional trypan blue test. Cells were resuspended in ice-cold 1∶1 mixture of growth medium and Matrigel Matrix (BD Biosciences) and 40–50 µl were injected subcutaneously into the flanks of 6–8-week-old mice. The primary tumor sizes were measured with a caliper on a weekly

basis and approximate tumor weights determined using the formula 0.5ab^2^, where b is the smaller of the two perpendicular diameters. All mice were sacrificed at the first signes of suffering or if the tumor measured ≥2 cm. Euthanasia was completed by CO2 inhalation following strict guidelines outlined by the panel on Euthanasia, American Veterinary Medical Association in SBU DLAR Facility.

### Subclonning and establishment of stable CR cancer cell lines

Since the ability to induce the round adherent colonies with smooth adges (holoclones) is associated with stem cells, upon appearance of such colonies, they were subcloned and propagated under defined stemness-promoting conditions, i.e. growing on type I collagen-coated surfaces in serum-free stem cell mediums, with repeated cell sorting, passaging as a 3D spheroids and NOD/SCID mice xenografts.

### Isolation, purification and characterization of the tumor-initiating cell phenotypes

To ensure more reliable isolation of CICs, cells were labeled with one or several common markers of stemness conjugated with different fluorescent dyes, including anti-human CD133/2-APC (clone 293C3) and CD133/1-PE (clone AC133; Miltenyi Biotec, CA, USA); CD166-PE (clone 105902; R&D Systems, MN, USA); CD44-FITC (clone F10-44-2), CD44-PE (clone F10-44-2; Invitrogen/Biosources, USA); CD44v6-FITC (clone 2F10; R&D Systems, USA), EpCAM-FITC (Biosource, CA, USA), Pan-Keratin (C11) -Alexa Fluor 488 (Cell Signaling) and all the isotype controls (Chemicon). Antibodies were diluted in buffer containing 5% BSA, 1 mM EDTA and 15–20% blocking reagent (Miltenyi Biotec) to inhibit unspecific binding to non-target cells. After 15 min incubation at 4°C, stained cells were sorted and analyzed with multiparametric flow cytometer BD FACS*Aria* (Becton Dickinson, CA). Alternatively, dissociated cells were centrifuged at 950 g for 5 min at 4°C, rinsed with sterile MACS buffer (Miltenyi Biotec, CA) and labeled with CD133 Abs directly or indirectly conjugated with ferromagnetic beads (Miltenyi Biotec, CA) as recommended by manufacturer.

### Immunocytochemical analysis of the pluripotency markers expression

For staining with OCT-4, Sox-2 and c-Myc, cells were fixed and permeabilized with Cytofix-Cytoperm kit (BD Pharmingen, San Diego, Ca, USA) according to the manufacturer's instructions. Cells were then incubated with monoclonal mouse IgG2b anti-human OCT-3/4, Sox-2 and c-Myc (Santa Cruz Biotechnology, Santa Cruz, Ca, USA) at 4°C for 30 minutes, washed twice with PBS and incubated with corresponding secondary Abs [phycoerythrin (PE)-conjugated polyclonal goat anti-mouse] at 4°C for 30 minutes. *h*EpCAM-FITC (Biosource, CA, USA), biotin-conjugated anti-human CD133 as primary Abs (Miltenyi Biotec, CA, USA) and streptavidin-FITC (BD Pharmingen, USA) as a secondary Abs were used.

### RNA-Seq

Libraries for NGS were generated using 1 microgram of total RNA. The RNA quality was assessed with an Agilent Bioanalyzer. All RNA samples had a RIN above 8. Sequencing libraries were generated using the Illumina TruSeq Stranded mRNA LT kit according to manufacturer recommendations. The quality of each library was evaluated with the Agilent bioanalyzer high sensitivity assay, and quantified by qPCR (Kappa Biosystem, CT). The libraries were pooled together based on the qPCR results, and then the pool was quantified again by qPCR. The pooled library was sequenced in one lane of a HiSeq2000 paired end 100bp flow cell. The Illumina HiSeq 2000 uses the sequencing-by-synthesis process, whereby in each cycle a single fluorescently tagged nucleotide is added to the clustered DNA templates, after which high-resolution images are taken to identify the nucleotide added. The added nucleotides are chemically modified so that they can accept another round of nucleotide addition, and the cycle is repeated. The HiSeq 2000 produces 100 bp sequences from one end of the DNA templates (single-end sequencing), and provides the option for generating additional sequence from the same templates from the other end (paired-end sequencing. Image processing and basecalling were performed as the runs progressed with Illumina's Real Time Analysis (RTA) software. The binary basecall files were streamed to a shared Linux server for further processing. The Illumina Casava pipeline (v1.8.2) was used to convert the binary files to fastq files containing the basecalled reads and per base quality scores. Only reads passing the standard Illumina quality filter were included in the output files. Casava was also used to de-multiplex reads into individual files for each barcode, allowing 1 mismatch in each barcode sequence.

### Western blotting

Nuclear extracts were prepared using Active Motif Nuclear Co-IP kit (Active Motif, Carlsbad, CA) according to manufacturer's protocol. The protein content was determined using BCA method and extract were suspended in Laemmli buffer at final concentration of 0.5 µg/ml. Cell pellets were suspended in Lysis Buffer (Active Motif, CA) and incubated for 10 min on ice on a rocking platform. After brief vortexing, cell lysates were centrifuged at 4,000 g for 30 min at 4°C. The protein content in the supernatant was determined by a Bradford method, and equivalent amounts of total proteins (10 µg) were resolved on 10% SDS-PAGE gel. After transferring to a polyvinylidene fluoride membrane, levels of various proteins were determined by Western blot analysis using antibodies specific for Oct4, Sox2, Nanog, Lin-28, c-Myc, GAPDH and β-actin, respectively (1∶500 dilution, 4°C, overnight). Following incubation with peroxidase-conjugated secondary Abs (anti-rabbit IgG; ECL, UK) for 1 hr at 25°C, blots were developed using the enhanced chemiluminesens (ECL) reagents. Alternatively, the blots were washed three times with PBST and incubated with AlexaFluor 680-conjugated goat anti-rabbit secondary antibody (Invitrogen) for 1 hr. Blots were then washed three times with PBST, twice with water, and the image captured on an Odyssey Infrared Imaging System (Li-Cor Biosciences).

### Drug Treatment

The CR4 cells were seeded onto the type I collagen-coated 96-well plates (500 cells/well), cultured in Stemline Pluripotent Stem Cell Culture media (Sigma) for 2 days, and the treatment was initiated upon ∼90% confluency. Paclitaxel and SBT-1214, 121602 and -12834 were dissolved in sterile DMSO and then serially diluted in serum-free media. Cells were treated for 48 hours and cell death was analyzed with the MTT assay [MTT  =  3-(4,5-dimethylthiazol-2-yl)-2,5-diphenyltetrazolium bromide] as recommended by manufacturer (Invitrogen).

### Statistical Analysis

The dose-response cytotoxicity of new-generation taxoids and paclitaxel was evaluated by standard MTT assay (all of the *in vitro* experiments were repeated at least three times and each measurement for the MMT assay was performed in 4 repeats). The dose-response points were plotted as a percentage of the untreated control, the absorbance of which was considered as 100%. Data were expressed as means ± SD for each drug concentration. Similarly, data from at least three different FACS analyses were also expressed as means ± SD for each marker. The statistical significance of differences was determined using the one-sided Students *t*-test. *P*<0.05 was considered statistically significant.
